# Mosquito repellence induced by tarsal contact with hydrophobic liquids

**DOI:** 10.1038/s41598-020-71406-y

**Published:** 2020-09-02

**Authors:** Hiroaki Iikura, Hiroyuki Takizawa, Satoshi Ozawa, Takao Nakagawa, Yoshiaki Matsui, Hiromi Nambu

**Affiliations:** 1 Material Science Research, Kao Corporation, 2-1-3 Bunka, Sumida, Tokyo 131-8501 Japan; 2 Material Science Research, Kao Corporation, 1334 Minato, Wakayama, Wakayama 640-8580 Japan; 3 Personal Health Care Products Research, Kao Corporation, 2-1-3 Bunka, Sumida, Tokyo 131-8501 Japan

**Keywords:** Wetting, Entomology

## Abstract

Mosquito legs have a unique highly water-repellent surface structure. While being beneficial to mosquitoes, the water-repellence of the tarsi enhances the wettability of hydrophobic substances such as oils. This high wettability induces strong attraction forces on a mosquito’s legs (up to 87% of the mosquito’s weight) towards the oil. We studied the landing behaviour of mosquitoes on oil-coated surfaces and observed that the mosquito contact time was reduced compared to that on hydrophilic-liquid-coated surfaces, suggesting that the oil coating induces an escape response. The observed escape behaviour occurred consistently with several hydrophobic liquids, including silicone oil, which is used globally in personal care products. As the repellent effect is similar to multiple hydrophobic substances, it is likely to be mechanically stimulated owing to the physical properties of the hydrophobic liquids and not due to chemical interactions. On human skin, the contact time was sufficiently short to prevent mosquitoes from starting to blood-feed. The secretion of *Hippopotamus amphibius*, which has physical properties similar to those of low-viscosity silicone oil, also triggered an escape response, suggesting that it acts as a natural mosquito repellent. Our results are beneficial to develop new, safe, and effective mosquito-repellent technologies.

## Introduction

Female mosquitoes transmit numerous infectious diseases. The global incidence of Dengue fever alone, borne by *Aedes* mosquitoes, has drastically increased owing to the expansion of the vector’s habitats, and the number of cases is estimated at 390 million per year^1,2^. The spread of these diseases can be triggered by multiple bites of a single mosquito. When a mosquito blood-feeds on an infected human, the infectious pathogen is ingested into her abdomen, and her next bite places an uninfected human at risk. Therefore, preventing mosquitoes from biting humans is an effective strategy against disease transmission.

The application of insect repellents plays an important role in protecting humans from insect bites^3,4^. Common strategies for repelling insects act on their olfactory senses mediated by volatile active agents and on their taste perception, exemplified as bitter tastants^5–9^. These dual mechanisms induce avoidance behaviour in mosquitoes. In addition to affecting the insect’s sense of smell and taste, DEET (*N,N*-diethyl-3-methylbenzamide) exhibits contact-based chemorepellence mediated by tarsal segments of the *Aedes* mosquito legs^10^. This multiple-mechanism action makes DEET particularly effective; it is the most widely used repellent, with its effects lasting for approximately six hours. However, to provide perfect protection from mosquito bites, a high-concentration DEET formulation must be applied carefully over the exposed skin. Moreover, several countries have imposed age-based restrictions on DEET, such as limiting the number of daily uses for children and infants^11,12^. Therefore, discovering additional mechanisms for repelling mosquitoes is important for the design of effective protection methods that could be safety used for all age groups. In this study, we explored a repellence mode that focuses on the unique physical properties of the surface of mosquito legs rather than their chemosensory neurons and receptors, because we expected that the wettability of liquids on the tarsi could be an important determinant factor for the motion of mosquitoes.

Mosquito legs are highly hydrophobic due to the fine geometrical structure of their surface (Supplementary Figs. S1a, b)^13,14^. This water-repellent nature generates a weight-supporting force on water surfaces; the maximum repulsive force of a single mosquito leg is 23 times the mosquito’s body weight. This allows female mosquitoes to use the surface as a foothold to lay their eggs and also permits the adult mosquitoes that emerge from the aquatic pupa to fly away from the surface (Supplementary Video S1). While the roughness of hydrophobic surfaces magnifies their non-wetting property with respect to water, it enhances their wettability with respect to hydrophobic liquids^15^. Thus, in comparison with a smooth surface, a hydrophobic rough surface is more susceptible to the adhesion of hydrophobic liquids, such as silicone oil and hydrocarbon-based oil. This induces a non-negligible attractive capillary force that will pull the surface of mosquito legs towards the liquid, which can have a significant impact on mosquito behaviour.

It has been reported that casting a silicone oil-based liquid on a water surface can inhibit the breeding of larvae and prevent female mosquitoes from staying on the treated surface to lay their eggs^16^; however, the study was primarily based on observations of the oviposition number of the eggs laid and does not focus on the physical properties of the liquid or its wettability to mosquito legs. In a preliminary study described below, we studied how the wetting of hydrophobic liquids affects the behaviour of mosquitoes landing on glass substrates coated with silicone oil (low-viscosity polydimethylsiloxane; L-PDMS). We measured the contact time of mosquitoes on substrates coated with different substances, and observed an escape response on oil-coated surfaces characterised by a short contact time. If this escape behaviour occurs consistently with different hydrophobic liquids, it could act an additional mosquito repellent mechanism. We then systematically investigated the relationship between the shortening of the contact time and the wetting of the hydrophobic liquids. We focused on the physical properties of the liquids such as surface tension and viscosity by comparing the wetting and mosquito contact time with medium- and high-viscosity polydimethylsiloxane (M-PDMS and H-PDMS, respectively) to that with L-PDMS. These liquids have very different viscosities owing to the different degrees of polymerisation, yet they have similar surface tensions because they all comprise the same repeating units (L-PDMS: γ = 19.2 mN/m, η = 0.0054 Pa s. M-PDMS: γ = 20.9 mN/m, η = 0.060 Pa s. H-PDMS: γ = 21.4 mN/m, η = 4.5 Pa s). In view of the possible existence of a mechanical stimulus in the mosquito legs to induce an escape response, we also examined the strength of the capillary force pulling the legs.

In addition to polydimethylsiloxane (a synthetic material), we investigated whether naturally occurring secretions could also affect insect behaviours based on their wettability towards mosquito tarsi. It has been reported that horse sweat contains hydrophobic proteins called latherin, which reduces the surface tension, and thus enhances wettability to the skin to improve thermoregulation^17^. In addition, the secretion of hippopotami (called red sweat) is an oily substance which protects against transdermal water loss^18^. Despite being blood hosts, hippopotami have no apparent protection method from insect biting, such as a long pelage or a coat patterning like zebras^19–22^. Therefore, we predicted that this exudate may provide protection against mosquitoes. To investigate this, we examined the wettability of the hippopotamus secretion on mosquito legs and assessed whether a substrate coated with this exudate affects mosquito behaviour.

## Results

### Contact angle of various liquids on the legs of mosquitoes

**Figure 1:**
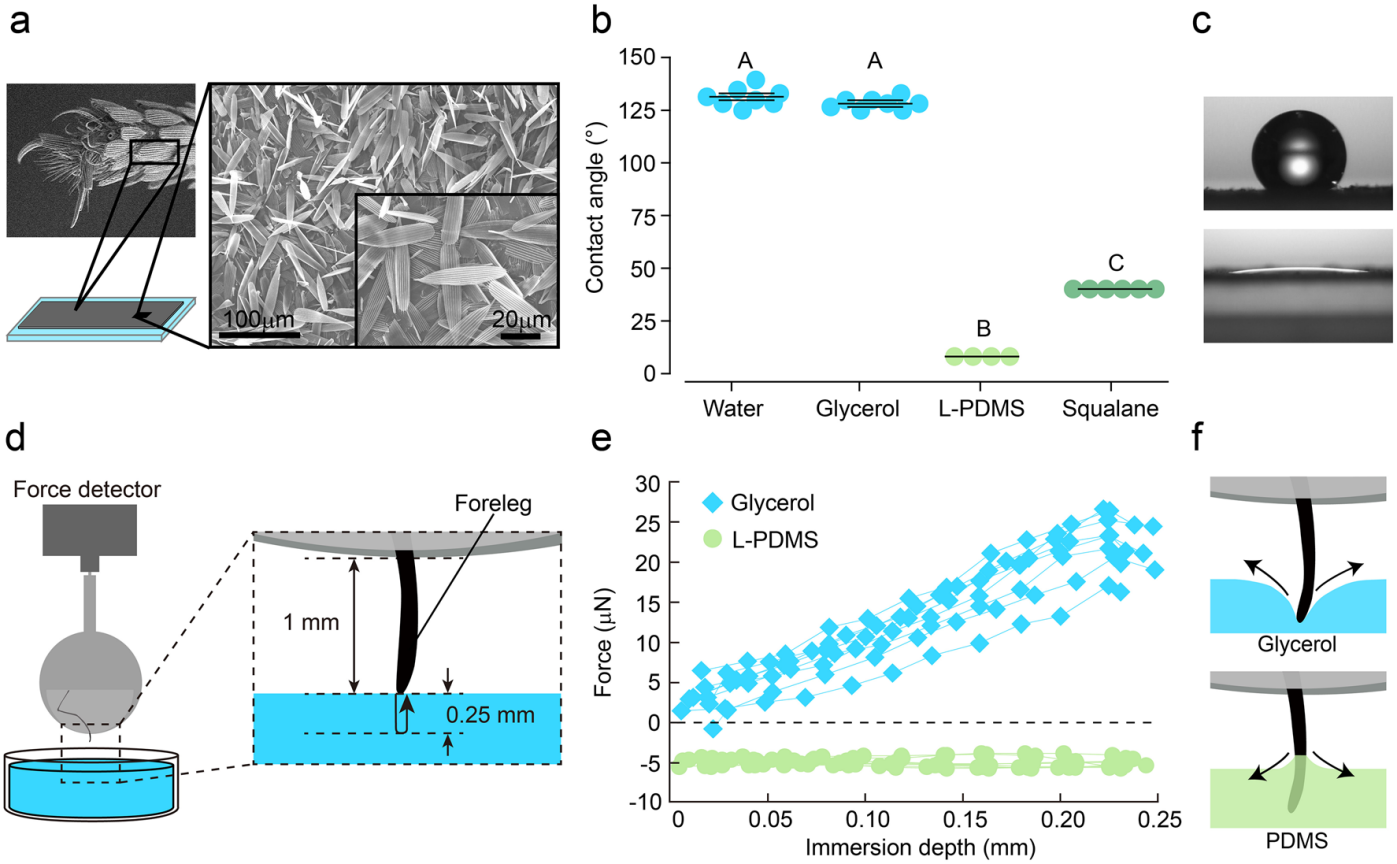
Hydrophobic liquids show high wettability with mosquito legs inducing attractive capillary force. (**a**) Mosquito-scale carpet. Collected scales of the legs are placed on double-sided tape. Scanning electron microscopy images of carpet surface (right). (**b**) Contact angle of droplets on the scale carpet at 1 s after liquid deposition (*n* = 4–8). Glycerol: surface tension (γ) = 65.0 mN/m, viscosity (η ) = 0.95 Pa s. Low-viscosity PDMS (L-PDMS): γ = 19.2 mN/m, η  = 0.0054 Pa s. Squalane: γ = 29.9 mN/m, η  = 0.042 Pa s. Water: γ = 71.8 mN/m, η  ~ 0.001 Pa s. Horizontal line represents the mean contact angle ± s.e.m. Different letters (A, B, or C) indicate significant variance among liquids (one-way analysis of variance (ANOVA) with the Tukey post hoc test, *P* = 10^–27^). The contact angle of each trial is displayed in Supplementary Table S2. (**c**) Droplets of glycerol (top) and PDMS (bottom) at 1 s after liquid deposition. (**d**) Schematic of mosquito leg contacting liquids. The foreleg is fixed on a force detector to obtain value for capillary force when immersed 0.25 mm into the liquids. (**e**) Force value with respect to the immersed depth of the forelegs (*n* = 8). Negative values mean an attractive capillary force pulling the leg into the liquids. (**f**) Schematics of mosquito tarsus immersion into glycerol and PDMS. Meniscus structure formed by wetting of PDMS causes an attractive capillary force.

To study the wettability of hydrophobic substances towards mosquito tarsi, we first determined the contact angle of various liquids on the mosquito legs. For a systematic contact angle measurement, we collected the scales that cover the surface of the legs and used them to prepare a carpet on which to deposit various liquids, instead of depositing the liquids directly on the legs. Scales from a total of 480 female mosquito legs were placed on double-sided tape (15 mm × 15 mm) affixed to a glass slide. Figure 1a shows scanning electron microscopy images of the carpet. We examined two hydrophobic liquids, low viscosity polydimethylsiloxane (L-PDMS, (C_2_H_6_OSi)_*n*_) and squalane (C_30_H_62_); a hydrophilic liquid, glycerol (C_3_H_8_O_3_); and water (Supplementary Table S1). L-PDMS, squalane, and glycerol are suitable for well-controlled experiments of wetting owing to their extremely low volatilities. Figure 1b shows the contact angle results. The droplets of glycerol were strongly repelled, similarly to the water droplets, as both liquids have similarly high surface tensions (Fig. 1c). The average water droplet contact angle was 130.8°, which is appreciably larger than that on any smooth solid surface (119°, derived from the surface of regularly aligned closed hexagonal packed –CF_3_ groups^23^). This illustrates the enhancement of non-wetting-liquid repellence by the rough surface of the legs. However, the L-PDMS and squalane contact angles were much smaller than 90°, indicating that the hydrophobic mosquito legs have a high wettability to hydrophobic liquids (Supplementary Table S2). We note that the L-PDMS droplets continued to spread on the surface after the measurements; thus, the equilibrium value of the contact angle is smaller than the measured value of 8.2°. In order to evaluate the wettability of L-PDMS on the mosquito legs, we determined the critical surface tension (γ_c_) of the mosquito legs by using the results of the contact angle measurements, because surface tension is an indicator of wettability (Supplementary Figs. S2a, b). The threshold value means that a liquid with a surface tension below γ_c_ spreads completely on the mosquito legs (contact angle, $\theta$ = 0°)^24^. The γ_c_ of the legs is 20.9 mN/m, indicating the L-PDMS (γ = 19.2 mN/m) shows total wetting on the mosquito legs.

### Attractive force measurements

**Figure 2:**
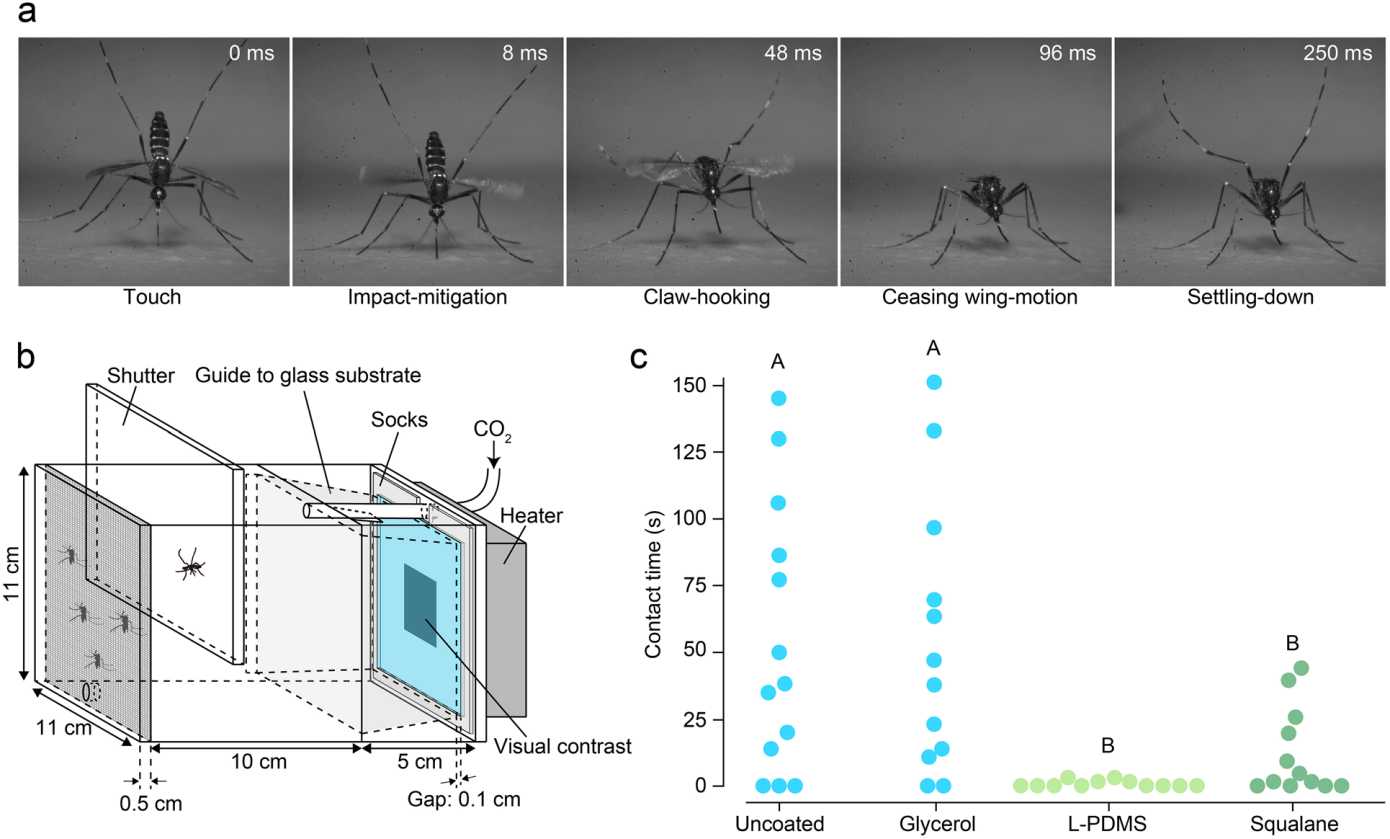
Wetting liquids trigger mosquito escape response. (**a**) Still video frame of mosquito landing. The mosquito immediately ceases wing-motion after contacting a substrate. (**b**) Schematic of contact-time assay. *Aedes albopictus* was used for all experiments. This chamber provides host cues (odour, heat, carbon dioxide, and visual contrast) to promote landing on ground-glass substrates. Applying liquids did not affect the attraction behaviour of mosquitoes to landing area (glass substrates) (Supplementary Fig. S4a). Surface roughness of liquid-coated substrates were identical (Supplementary Fig. S5a). (**c**) Contact-time of mosquitoes on liquid-coated glass substrates (*n* = 12–13). Application ratio: 0.25 mg/cm^2^. Different letters (A or B) indicate significant variance among liquids (one-way ANOVA with the Tukey post hoc test, *P* = 6.77 × 10^−4^). The contact time of each trial is displayed in Supplementary Table S3. See also Supplementary Video S3.

We then measured the force generated by contact between the mosquito tarsi and L-PDMS or glycerol. The measurement set-up is shown in Fig. 1d. The mosquito’s foreleg was fixed to the force detector. The vessel containing the liquid was raised such that the leg approached the liquid surface from above and was then immersed in the liquid. The obtained force value with respect to the immersed depth is plotted in Fig. 1e. In the case of glycerol, the force is zero when the tarsus just reaches the liquid surface and then becomes repulsive due to the formation of a meniscus with convex curvature, as schematically shown in the upper part of Fig. 1f. The repulsive force then grows as the immersion depth increases owing to the steeper liquid surface surrounding the leg^13,14^. On the other hand, in the case of L-PDMS, a large attractive force of 4.96 ± 0.52 $\upmu$N (± s.d.) is applied to the foreleg due to the formation of a meniscus with concave curvature, as schematically shown in the lower part of Fig. 1f^25^. The obtained value of this capillary force is almost constant at each immersion depth, indicating that the change in contact angle vanishes during the withdrawal process. Mosquitoes generally use a total of four legs (forelegs and middle legs) in landing (Fig. 2a, Supplementary Video S2). Hence, our results suggest that a landed mosquito is subject to a maximum attractive force of ~ 87% of the mosquito’s weight in magnitude (average weight of tested mosquitoes: 2.33 mg, *n* = 80).

### Behaviours of mosquitoes landing on hydrophobic liquids

In our preliminary study, we measured the duration that female adults stay on substrates coated with different liquids. As schematically shown in Fig. 2b, we prepared a chamber providing host cues such as odour, heat, carbon dioxide, and visual contrast to promote landing on a liquid-coated ground-glass substrate, and measured the tarsal contact time of female mosquitoes on the substrate (Supplementary Figs. S1c, S3)^26–30^. We examined four cases: 1) no coating, 2) glycerol coating, 3) L-PDMS coating, and 4) squalane coating. The results are shown in Fig. 2c (Supplementary Table S3). The results with no coating are similar to the results with glycerol. The contact time varied widely between 0.018 and 151 s, and various optional behaviours were observed, such as immediately flying away, walking around to find a blood source, and staying stationary for a long time. In comparison, in the cases of L-PDMS and squalane, the hydrophobic coatings affected the contact time, especially for L-PDMS; the mosquitoes did not stay on the L-PDMS-coated surface longer than 3.0 s. (Supplementary Video S3). A high-speed camera was used to record the contact between the leg of a flying mosquito and the L-PDMS coating. The video showed that the liquid in the vicinity of the contact area wetted the legs, resulting in the formation of a concave meniscus structure (Supplementary Video S4).

### Effect of PDMS viscosity and application ratio on contact time

**Figure 3:**
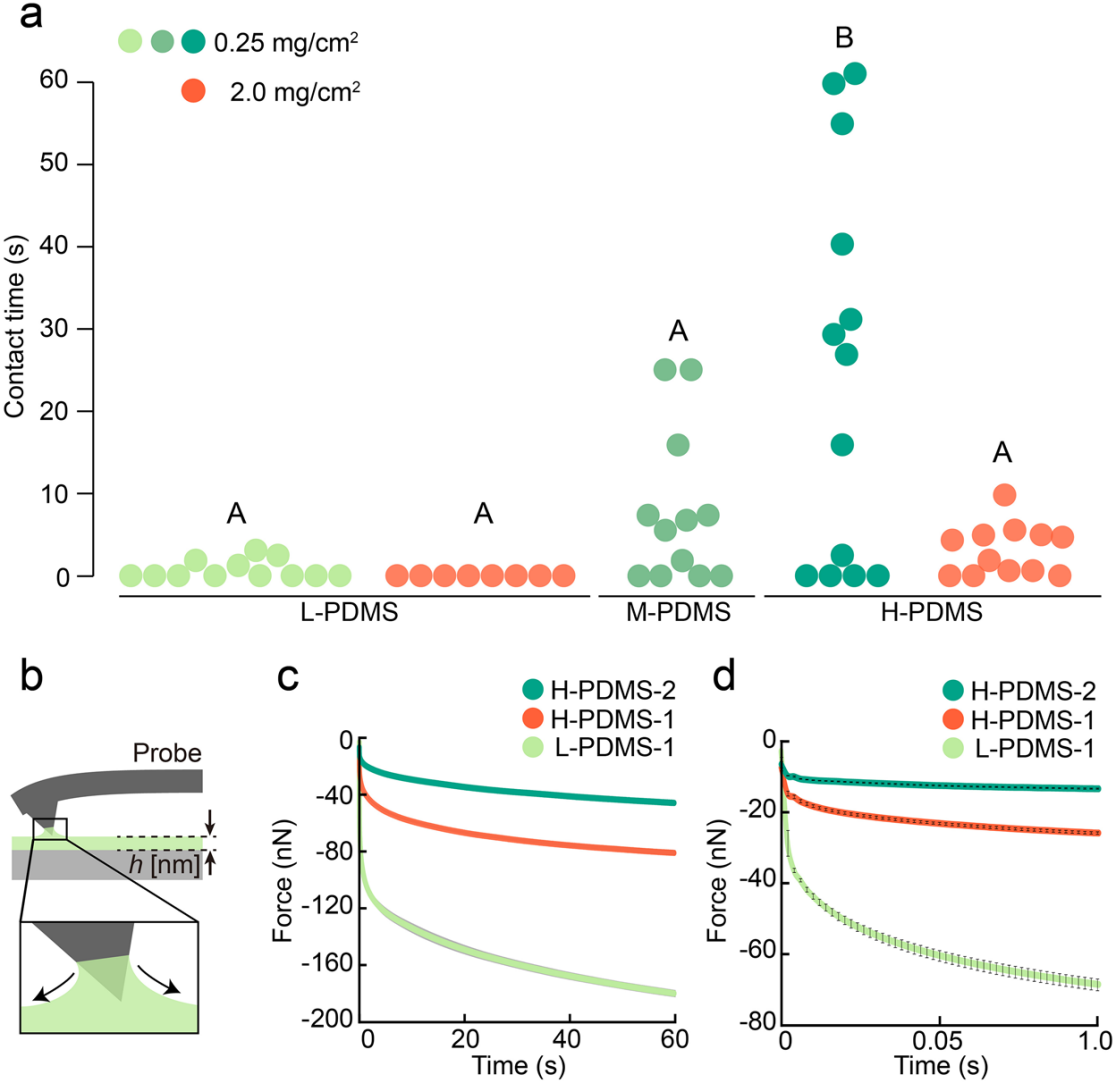
Rapid wetting on mosquito legs distract staying on liquid-coated substrates. (**a**) Contact-time of mosquitoes on glass substrates coated with PDMSs (*n* = 8–13). L-PDMS (γ = 19.2 mN/m, η  = 0.0054 Pa s) (light green and orange circle), M-PDMS (γ = 20.9 mN/m, η  = 0.060 Pa s) (green circle), and H-PDMS (γ = 21.4 mN/m, η  = 4.5 Pa s) (dark green and orange circle). Application ratio: 0.25 and 2.0 mg/cm^2^. Liquid viscosity difference did not affect attraction behaviour of mosquitoes and coated state on the substrates (Supplementary Fig. S4b, S5b). Different letters (A or B) indicate significant variance among liquids (one-way ANOVA with the Tukey post hoc test, *P* = 2.27 × 10^−5^). The contact time of each trial is displayed in Supplementary Table S3. (**b**). Schematic of AFM-probe contacting on a PDMS film. Attractive capillary force caused by meniscus formation is obtained with atomic force microscopy. (**c**) Dynamical generation of attractive capillary force between the probe and PDMS films (*n* = 5–6). LPDMS-1 (thickness, *h* = 200 nm), HPDMS-1 (*h* = 200 nm), HPDMS-2 (*h* = 120 nm). Data are plotted as the mean value of the attractive force ± s.d. (**d**) Attractive capillary force in short time range of (**c**). Data are plotted as the mean ± s.d.

To better understand how the physical properties of the hydrophobic coatings affect the mosquito contact time, we examined low-viscosity PDMS (L-PDMS), medium-viscosity PDMS (M-PDMS), and high-viscosity PDMS (H-PDMS). PDMS is convenient for conducting this experiment, as its viscosity is adjustable depending on the molecular weight, without significantly affecting the surface tension or equilibrium contact angle on mosquito legs (Supplementary Table S1). Figure 3a shows the effect of viscosity on the tarsal contact time; all tested mosquitoes left the L-PDMS-coated surface in under 3 s, but it took up to 61 s for some mosquitoes to leave the H-PDMS-coated surface.

In addition to the dependence on liquid viscosity, increasing the application amount from 0.25 to 2.0 mg/cm^2^ reduced the tarsal contact time, although the surfaces of the coated substrates were chemically identical. With the thicker H-PDMS film, the maximum time that the tested mosquitoes stayed on the surface was shortened from 61 to 10 s, and in the case of L-PDMS, the time was shortened from 3.0 to 0.18 s. We note that two patterns occurred in the mosquito contact process: flying away without ceasing wing-motion immediately after touching the glass substrate (Supplementary Video S5), and settling down then flying away (Supplementary Video S3). All the video recordings of mosquitoes touching the coated substrates were analysed to examine whether the mosquitoes ceased flapping to land on the substrate. The results were summarised for each liquid sample and the ratio of mosquitoes that made ceasing wing motions after making contact with the substrate was calculated. Notably, the ratio of ceasing wing-motions was 0% for 2 mg/cm^2^ L-PDMS, compared to 58.3% for the same coating thickness of H-PDMS; hence, all mosquitoes initiated an escape response immediately after tarsal contact with the L-PDMS-coated surface with high application amount (Supplementary Table S3).

### Effect of PDMS viscosity on attractive capillary forces

We evaluated the dependence of PDMS viscosity on the generation of capillary forces caused by the wetting behaviour using atomic force microscopy (AFM); a force tensiometer could not discern the different dynamics of wetting owing to the use of vessels containing a large quantity of liquid. We prepared a thin film of PDMS and measured the capillary force upon contact between the AFM probe and the liquid (Fig. 3b)^31^. We examined three oil-film cases: 1) L-PDMS1 (film thickness, *h* = 200 nm), 2) H-PDMS1 (*h* = 200 nm), and 3) H-PDMS2 (*h* = 120 nm) (Supplementary Table S4). We investigated the effect of viscosity on the capillary force with the two equal-thickness films of L-PDMS1 and H-PDMS1, and the effect of the application ratio on the capillary force by using the H-PDMS1 and H-PDMS2 films with different thicknesses. Figure 3b shows a schematic of the capillary force caused by meniscus formation during the measurement.

The results are shown in Fig. 3c, and the part of the curve between 0 and 1 s is magnified in Fig. 3d. In the case of L-PDMS, rapid meniscus formation due to wetting generated a capillary force in a short time (within 1 s). Additionally, from the comparison between the two H-PDMS films, we can see that thicker film increased the speed of capillary force generation. Comparing the results with L-PDMS and H-PDMS in the above two experiments, we can observe that there is a correlation between the maximum mosquito contact time and the liquid viscosity; rapid meniscus formation leads to a short maximum contact time while slower meniscus formation leads to a longer maximum contact time.

### Behaviours of mosquitoes landing on actual human skin

**Figure 4:**
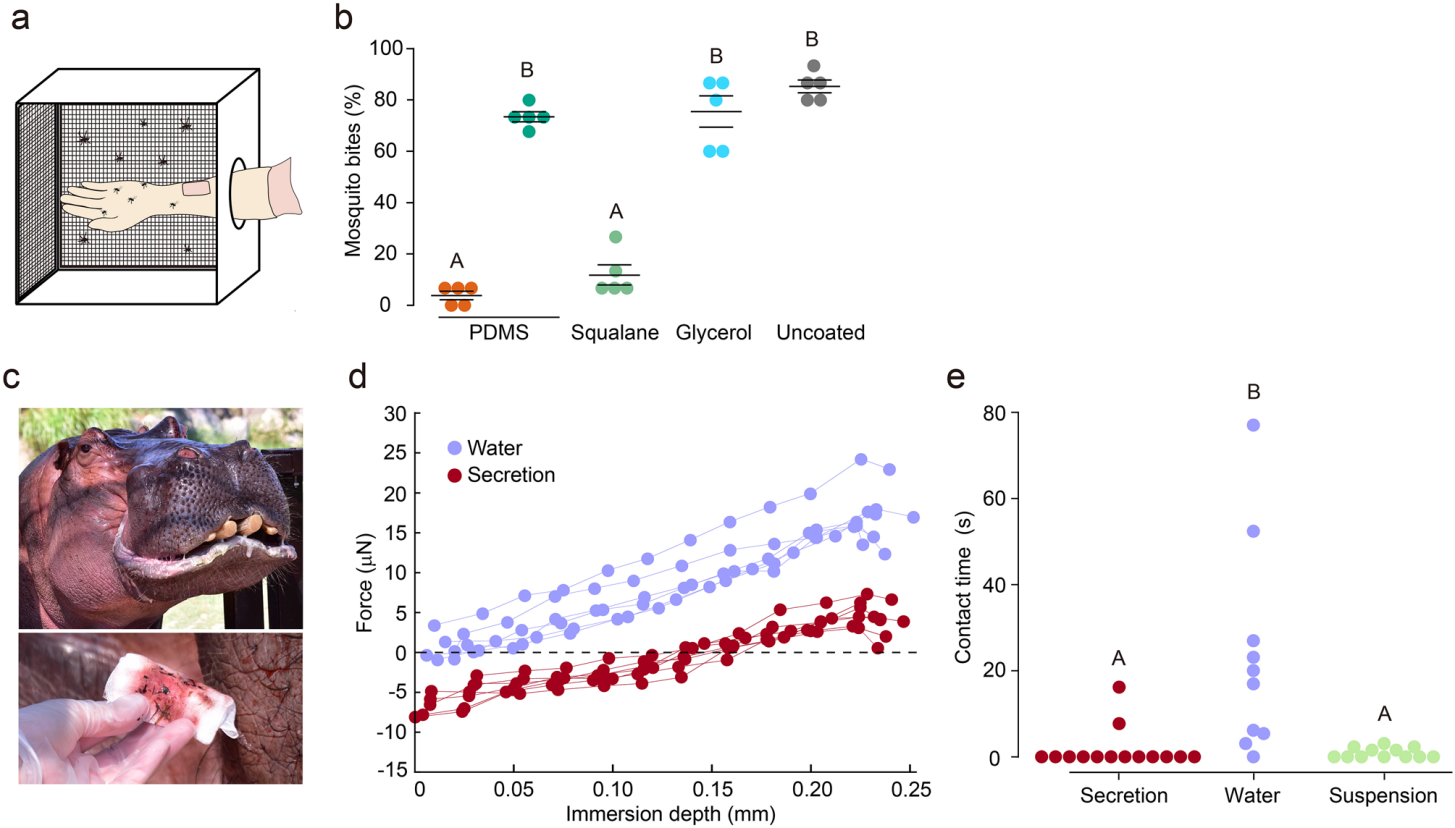
Wetting-liquid-based repellent is effective on human and hippopotamus skin. (**a**). Diagram of mosquito biting assay. Exposure area of forearms is 4 cm × 5 cm. (**b**) Mosquito bites on liquid-coated forearms. L-PDMS (orange circle) and H-PDMS (dark green circle). Application ratio: 0.25 mg/cm^2^. Horizontal line represents the mean ± s.e.m. (*n* = 5). Different letters (A or B) indicate significant variance between liquids (one-way ANOVA with the Tukey post hoc test, *P* = 10^–13^). (**c**) *Hippopotamus amphibius* male born in 1982 and red sweat. (**d**) Force value with respect to the immersion depth of mosquito forelegs. The value is obtained with the experimental setup used in Fig. 1e (*n* = 6–7). Hippo secretion (dark red circles), water (purple circles). The experiment with the hippo secretion was conducted by pouring ~ 0.5 mL into a small plastic petri dish. (**e**) Contact-time of mosquitoes on ground glass substrates coated with liquids (*n* = 10–14). Application ratio of hippo secretion and water: 2.0 mg/cm^2^. Applying liquids did not affect the attraction behaviour of mosquitoes to the landing area (glass substrates) (Supplementary Fig. S4c). Application ratio of silica nanoparticle suspension in L-PDMS: 0.25 mg/cm^2^. Hippopotamus secretion: γ = 25.1 mN/m. Suspension: γ = 18.9 mN/m. The letters (A or B) indicate the significant variance between the different liquids (one-way ANOVA with the Tukey post hoc test, *P* = 4.37 × 10^−4^). The contact time of each trial is displayed in Supplementary Table S3.

We performed an experiment to investigate whether an oil coating could trigger mosquito repellence on actual human skin. For this experiment, we conducted a mosquito biting assay wherein the forearms of human volunteers were topically applied with the liquids used in the contact time tests and then inserted into an insect cage. The measurement procedure is shown in Fig. 4a. We counted the number of mosquito bites as a ratio of the total number of landed mosquitoes. The results are shown in Fig. 4b. In the case of uncoated skin, about 85% of the landed mosquitoes led to bites. This ratio was greatly reduced for the squalane- and L-PDMS-coated skin; for L-PDMS, only 4% of the landed mosquitoes engaged in biting behaviour. These results are consistent with the previous experiments; L-PDMS and squalane, which reduced the contact time on glass substrates, distracted the mosquitoes from biting actual human skin, whereas H-PDMS and glycerol did not effectively repel them.

### Behaviours of mosquitoes landing on hippopotamus secretion

We collected secretion samples from *Hippopotamus amphibius* by wiping the face of the animal, and evaluated the surface tension (γ_secretion_) and viscosity of the secretions (Fig. 4c). Even though the liquid is water-based (water content: 97.25%), the surface tension was considerably low (γ_secretion_ = 25.1 mN/m) and similar to that of PDMS and squalane. The value satisfies γ_c_ < γ_secretion_ < γ_squalane_; therefore, the liquid partially wets mosquito legs at the interface. The wettability was also revealed by the results obtained with the force tensiometer (Fig. 4d); a large attractive force was generated during the withdrawal process of the immersed tarsi from the secretion, which is comparable in magnitude to that for L-PDMS. We notice that the force was positive when the immersion depth was large, and it gradually decreased and became negative as the immersion depth decreased. This is because the contact angle of the secretion is not determined as a unique value owing to the effect of contact angle hysteresis derived from physical and chemical heterogeneities of the liquid/solid interface, such as the rough surface of the mosquito legs and surface segregation of some ingredients of the secretion^32^. In addition, the secretion showed shear-thinning behaviour that was strongly dependent on the shear rate; although the viscosity of the secretion was low (comparable to that of L-PDMS) at shear rates of ≳10^2^ s^−1^, the viscosity was larger than that of H-PDMS at lower shear rates (Supplementary Fig. S6).

To investigate whether a liquid with shear-thinning behaviour affects the tarsal contact time, we prepared a silica nanoparticle suspension in L-PDMS, because mixing a fine powder into a liquid can increase its viscosity at low shear rates. This is due to the formation of a network structure of the powder in the liquid. The flow curve of this suspension was similar to that of the secretion (Supplementary Fig. S6). We conducted mosquito contact-time tests with the secretion, the silica nanoparticle suspension, and distilled water by employing the chamber described above (Fig. 2b). Like the L-PDMS coating (Fig. 2c), the secretion coating reduced the contact time, whereas the distilled water coating did not effectively shorten the contact time (Fig. 4e). In addition, the silica-particle suspension in L-PDMS reduced the contact time to a similar degree as L-PDMS, indicating that shear-thinning fluids can induce mosquito repellence. The results for this contact time measurement indicate the possibility that in mosquito habitats, hippopotamus secretion behaves as a coating with low surface tension and low viscosity during mosquito landing, and thus acts as an insect repellent that takes advantage of this rather simple method, in addition to its role as a moisturiser, antiseptic, and sunscreen^18,33–35^.

## Discussion

We investigated the possibility of mosquito repellence based on the physical properties of hydrophobic liquids. We found that applying hydrophobic liquids to a surface induces an escape response of mosquitoes after tarsal contact with the liquid-coated surface. Significant shortening of the contact time occurred consistently when hydrophobic liquids were applied. With polydimethylsiloxane, which is widely used in personal care products, the contact time could be sufficiently shortened to prevent mosquitoes from biting human skins. L-PDMS was the most effective hydrophobic repellent among those explored in this study.

The physical properties of the rough water-repellent mosquito legs and the wetting behaviour of the hydrophobic liquids indicate that the escape response is triggered by meniscus formation of the wetting liquids. There was a strong correlation between the maximum contact time and the viscosity of the wetting liquids (Fig. 3a); the maximum contact time is sensitive to the speed of meniscus formation. This strong correlation suggests that the shortening of the contact time occurs due to a mechanical phenomenon; we expect it is related to the capillary force of the wetting liquids. It is possible that the longer maximum contact time on the H-PDMS-coated surface (compared to the L- and M-PDMS-coated surfaces) occurs, because it takes longer to generate the attractive capillary force required for repellence with a higher viscosity liquid; these liquids all have similar surface tension, suggesting that the force exerted by them would be equivalent when the same size meniscus was formed on the legs. AFM measurements support this as a possible cause; the lower the viscosity or thicker the liquid film, the more rapidly attractive forces are generated by meniscus formation (Fig. 3c). Moreover, even though the tested PDMSs have the same siloxane skeleton linked to methyl groups, the maximum contact time varies depending on the viscosity and the amount of liquid applied, suggesting that the shortening of the time is not induced by a chemical property of the liquids. We note that the maximum contact times of the M- and H-PDMS cases with an application amount of 0.25 mg/cm^2^ appear to act as sharp upper limits for the mosquito contact time (see Fig. 3a), again suggesting the existence of a mechanical stimulus in the mosquito’s legs which triggers an escape motion, even though the force is not enough for a mosquito to be trapped.

Mosquitoes deliberately manoeuvre their unique long tarsi to minimise take-off impact, which reduces the chance that their landing is detected by the host^36^. Hence, maintaining fine foot manipulation may be essential after landing on a blood host. It has been reported that *Drosophila* flies have evolved somatosensory neurons in their legs to enable them to respond to external mechanical stimuli^37,38^; moreover, the leg proprioceptor of *Drosophila* senses the tibia position and movement^39^. It is therefore likely that mosquitoes have also evolved developmental sensory systems in their legs. Further detailed experimental investigations are necessary to clarify the connection between the response behaviour and the capillary force, including experimentally evaluating the dynamics of wetting on actual mosquito tarsi in contact with the wetting liquids.

We investigated hippopotamus secretion as a possible natural mosquito repellent with a similar strategy based on the wetting of hydrophobic liquids. We collected the secretion from the face of a living hippopotamus and measured the mosquito contact time on secretion-coated glass substrates (Fig. 4e). We found that the secretion indeed shortened the mosquito contact time. To analyse the results, we measured the shear rate dependence of the viscosity of the secretion and found shear-thinning behaviour; the viscosity was large (comparable to that of H-PDMS) at low shear rates, and decreased as the shear rate increased. When the shear rate exceeded 10^2^ s^−1^, the viscosity was similar to that of L-PDMS. In the contact time experiments, we calculated the ratio of mosquitoes exhibiting ceasing wing motions after making contact with the coated surface. This ratio was 21.4% for the secretion-coated substrates, which is much closer to the ratio for the L-PDMS-coated substrates than that for the H-PDMS-coated substrates (Supplementary Table S3). This indicates that the escape behaviour is associated with the higher-shear-rate regime of the liquids, suggesting the dynamic nature of the repellence process.

For practical application as a mosquito repellent, the flow and protection duration of the liquid substance on actual human skin must be considered. Due to its low viscosity, pure L-PDMS is likely to flow and thin when coated on human skin as time progresses. The hippopotamus secretion had high viscosity at low shear rates, which is likely to limit the flow of the secretion over the hippopotamus’s skin, thus ensuring that it covers the exposed skin for a longer time. Considering the benefits of a liquid with shear-thinning behaviour for both application and repellence, we prepared a suspension of fine silica powder in L-PDMS to simulate the shear thinning behaviour of the hippopotamus secretion (see Supplementary Fig. S6). The suspension had a large viscosity at low shear rates and a low viscosity close to that of pure L-PDMS at high shear rates. The results of mosquito contact time tests with this suspension showed that it had a similar repellent effect to that of pure L-PDMS (Fig. 4e).

To summarise, in addition to the conventional mosquito repellents, a new, safe, and effective mosquito repellent is possible by utilising the widely used polydimethylsiloxane.

## Methods

### Mosquitoes

*Aedes albopictus* eggs were purchased from Sumika Technoservice Corporation. Mosquitoes were reared in microclimate chambers at 28 °C and a controlled relative humidity (RH) of 70% with a 12 h dark/light cycle. Eggs were hatched in distilled water and larvae were fed TetraMin Baby. Pupae were moved in a small cup with distilled water, and the cup was placed in an insect cage with dimensions of 30 cm × 30 cm × 30 cm (BugDorm-1, MegaView Science Co., Ltd.). Adult mosquitoes were mated in the insect cage for 1 week. Mosquitoes were fed a 10 wt% sucrose solution. All experiments were conducted with 7- to 14-day old mosquitoes.

### Materials

Silicone oils (PDMS), KF96A 6cs (L-PDMS), KF96 50cs (M-PDMS), and KF 96-5000cs (H-PDMS) were purchased from Shin-Etsu Chemical Co., Ltd. Squalane and glycerol were obtained from FUJIFILM Wako Pure Chemical Corporation. The surface tension of each substance was measured using the Wilhelmy plate method using a platinum plate at 25 °C (Tensiometer K100, KRÜSS Optic GmbH). Viscosities were measured using a rotary viscometer (Viscometer TVB-10, Toki Sangyo Co., Ltd.) with an M2 rotor (rotation speed: 12 rpm) at 23 °C, except for measurements of L-PDMS and H-PDMS (η = 0.0054 and 4.5 Pa s). The viscosity of H-PDMS was measured using the same rotor at 6 rpm, while the viscosity of L-PDMS was measured using an L-adaptor rotor (rotation speed: 30 rpm). DEET was purchased from Tokyo Chemical Industry Co., Ltd. The surface tension was measured using the Wilhelmy plate method using a platinum plate at 25 °C. The viscosity was measured using a rotary viscometer and an L-adaptor (rotation speed: 30 rpm) at 23 °C.

To prepare a liquid sample that had viscosity, shear-thinning behaviour, and surface tension comparable to hippopotamus secretion, hydrophobic fumed silica (Aerosil R972, Evonik) was prepared, because this substance was able to add shear-thinning behaviour to PDMS. A 3 wt.% fumed silica suspension in KF96A 6cs (L-PDMS) was prepared. To evaluate the shear-thinning behaviour, viscosity was measured using a rheometer (MCR302, Anton Paar) as a function of shear rate (flow curve). CP50 parallel plates were used for the measurements ($\phi$ = 49.969 mm, angle: 0.994°). The flow curves resulting from three separate measurements were highly similar. The surface tension of the suspension was measured using the Wilhelmy plate method using a platinum plate at 25 °C.

The ground-glass substrates (dimensions: 80 mm (length) × 80 mm (width) × 2 mm (thickness)) used in mosquito-contact-time experiments were purchased from Ookabe Glass Hd. We used a rough-surfaced substrate as mosquitoes cannot land on smooth surfaces because their claws cannot find any foothold. The surface roughness (arithmetical mean height) of the substrates was 3.52 $\upmu$m (Supplementary Fig. S5c), and we confirmed that mosquitoes were able to land on the uncoated substrates. Skin replica substrates (No. 10C, H010C-021) used in high-speed camera recordings of mosquito landings (Fig. 2a) were purchased from Beaulax Co., Ltd. The polyurethane disc-shaped substrates (diameter: 5.0 cm) were replicates of the cheek of a 20-year-old woman.

### Mosquito contact-time tests

We used an acrylic chamber with dimensions of 16 cm (length) × 11 cm (width) × 11 cm (height) (Fig. 2b). A ground-glass substrate was fixed on the centre of an acrylic plate with dimensions of 11 cm (length) × 11 cm (width) × 0.5 cm (height). To attract mosquitoes to the glass substrates, socks, a heater, and an air tube were placed in the chamber. The socks had been worn by a human for 10–12 h to collect odour, and the sole area was cut with scissors to place around the glass substrates. A heater (LVPU-70, VICS) was set behind the glass substrate and its surface was maintained at 32–33 °C. The pre-set temperature of the heater was 38 °C. An air tube expelled humidified air supplemented to a concentration of 20% carbon dioxide into the chamber (flow rate, 1.5 L/min). Humidified room air was carbon-filtered (SUPELPURE HC 2–2,445-U, Supelco) by using an air pump (MP-Σ300NII, Shibata Scientific Technology Ltd.) and mixed with carbon dioxide gas flowed by another pump (Digital Mass Flow Controller, Azbil Corporation) in the front gas chamber. Between the glass substrate and the acrylic plate, a square sheet of black paper measuring 3 cm × 3 cm was inserted to promote mosquito contact on the centre of the glass substrate.

A total of five female mosquitoes were loaded into an anteroom with dimensions of 10 cm × 10 cm × 10 cm. These mosquitoes were fasted for 8–12 h with a water source prior to contact-time experiments. After an acclimation period of 2 min, the partition was opened, and the mosquitoes were given access to a ground-glass substrate coated in liquid. The liquid samples were applied to the substrates by hand using latex gloves. To record the contact time of mosquitoes that had never touched the substrate, only the mosquito that was first attracted to the substrate was filmed. If no mosquitoes were attracted to the glass substrates within 3 min after the partition was opened, the video was discarded. After each trial, the mosquitoes were replaced with five new specimens. Contact time was defined as the elapsed time between the recorded time when at least one tarsi or proboscis contacted the substrate and the time when all mosquito tarsi were removed (Supplementary Fig. S3). When a mosquito stayed on the substrate for a short time (contact time: < 10 s), a high-speed camera (Mini UX, Photon Ltd.) was used to obtain videos. The camera had a light source emitting a wavelength of 850 nm to reduce the chance that the mosquitoes would detect the light and react during image capture^40^. The shutter speed was 500 frames per second (fps), and a flash was produced at the moment of image capture. Because the camera cannot record over a long time, a video camera (HDR-CX480, Sony Corporation) was additionally used to record mosquito contact behaviour when the mosquito stayed for more than 10 s. Moreover, all the video recordings of mosquitoes touching the coated substrates were analysed to examine whether the mosquitoes ceased flapping to land on the substrate. The results were summarised for each liquid sample and the ratio of mosquitoes that made ceasing wing motions after making contact with the substrate was calculated. The number of contacts on the glass substrate corresponds to the number of video recordings of the experiment on a given liquid sample. The experiments were performed at 28 °C and 65% RH. In experiments using hippo secretion and distilled water (Fig. 4e), the heater was set to 30 °C, and the surface of the glass substrate was maintained at 29 °C to reduce the evaporation of the liquids.

### Attraction rate of mosquitoes to ground-glass substrates coated in liquids

The attraction rate measurement was conducted in the acrylic chamber. However, the front area of the ground-glass substrate was sloped, and there was no space for mosquitoes to remain after being attracted by a host cue; therefore, the length of this area was changed from 5 to 10 cm. The position of the slope was not changed. In addition, a mesh was attached to the front of the glass substrate to prevent mosquitoes from contacting the liquid directly. A total of 15 mosquitoes were injected into the chamber to increase the accuracy of the attraction rate. After an acclimation period of 2 min, the partition was opened, and mosquitoes were given access to a ground-glass substrate area. After sending the host cue for 3 min, the shutter was closed. The attraction rate was calculated from the number of mosquitoes flying into the side where the glass substrate was present.

### Contact angle measurement on a mosquito-scale carpet

Female mosquitoes were anaesthetised by placing them on wet ice and all legs were removed with tweezers. A total of 60 legs were placed on double-sided tape (No. 5000NS, Nitto Denko Corporation) (tape dimensions: 15 mm × 15 mm) on a glass slide (S1111, Matsunami Glass Ind., Ltd.), then the legs were rolled on the tape to collect the scales (Fig. 1a). Subsequently, the bare legs were removed from the surface of the double-sided tape. This was repeated eight times and 480 legs (approximately 80 female mosquitoes) were used to prepare the scale carpet on the area. This carpet was observed using a scanning electron microscope (JSM-IT500HR, JEOL Ltd.).

Droplets (3 $\upmu$L) of L-PDMS, glycerol, water, squalane, and DEET were deposited on the scale carpet, and the contact angle was measured after 1 s using a contact angle meter (Drop Master, Kyowa Interface Science Co., Ltd.). As the droplets of glycerol, water, and DEET did not significantly spread on the scale carpet, we prepared a 2.5 mm × 15 mm carpet to measure the contact angle effectively. As the smaller carpet was one-sixth the area of the 15 mm × 15 mm carpet, only 80 legs were used to prepare it. In the silicone oils and squalane experiments, the mosquito carpet was replaced after each trial because the liquid significantly spread. In the case of glycerol, water, and DEET, any part of the carpet that the liquid partially wet was not used for measuring the contact angle again.

### High-speed camera recording of mosquito landing on human skin replica

We prepared a cuboid acrylic box with internal dimensions measuring 6 cm. Four skin replicas were vertically fixed to the interior sides of the box with double-sided tape (No. 5000NS, Nitto Denko Corporation). After introducing mosquitoes into the box using an aspirator, we provided slight disturbance by lightly bumping the box on a table to cause the mosquitoes to fly into the air and alight on one of the replicas (Fig. 2a). The moment when the mosquito alighted on the skin replica was recorded using a high-speed camera (Mini UX, Photon Ltd.). The shutter speed was 1,000 fps, and a flash was produced at the moment of image capture.

### High-speed camera recording of tarsi connection with the silicone oil surface

A skin replica covered with M-PDMS (η = 0.060 Pa s; application ratio: 2 mg/cm^2^) was fixed vertically on a wall. We then prepared a small polystyrene box (No. 2, 2,262, Sanplatec Corporation) with dimensions of 5.5 cm (length) × 1.4 cm (width) × 3.7 cm (height). To make an opening, one of the lateral sides of the box was removed. Four mosquitoes were placed into the box using an aspirator and the opening was placed towards the surface of the skin replica affixed to the wall. To attract the mosquitoes to the skin replica, its surface temperature was maintained at 32 °C using a heater. A high-speed camera (Mini UX, Photon Ltd.) was used to record the contact between the leg of a flying mosquito and the L-PDMS coating. The shutter speed was set to 1,000 fps, and a flash was produced at the moment of image capture.

### Obtaining surface profiles for ground-glass substrates.

Surface images of the ground-glass substrates were taken using a confocal laser microscope (VK-9700, Keyence Corporation). This device was able to image the surface of the substrates by reflectance measurement using a confocal optical system. PDMS, glycerol, and squalane were applied to the ground-glass substrates (application ratio: 0.25 or 2.0 mg/cm^2^) and their coated and uncoated surfaces were imaged. The surface roughness (arithmetical mean height) was calculated using the surface images of the ground-glass substrates (*n* = 3).

### Force measurement upon mosquito tarsi contact with liquids

A force tensiometer (Tensiometer K100, KRÜSS Optic GmbH) was used to detect the force when mosquito tarsi contacted the liquids. The forelegs of mosquitoes were attached to a metal cylindrical plate with double-sided tape (No. 5000NS, Nitto Denko Corporation), such that the tip of the tarsus protruded 1 mm (Fig. 1d). Subsequently, the same size double-sided tape was stacked from above to fix the legs. A metal plate was placed on the tensiometer; the liquid stage approached at 6 mm/min until its deposition was detected, then the leg was immersed 0.25 mm into the liquid at 2 mm/min. Next, the force of the withdrawal process at the same speed was recorded in 20-$\upmu$m steps. The fixed foreleg was replaced after each force measurement, because the sample might be contaminated by contact with the liquid. The liquid deposition was evaluated when the detector measured a load of 0.1 mg. An experiment was conducted by pouring approximately 60 mL of L-PDMS, glycerol, or water into a glass petri dish. For the experiments with the hippopotamus secretion, we used a small polystyrene dish (Iwaki, Microplate with Lid, 3,860–096, Asahi Glass Co., Ltd.) into which we poured 0.5 mL of the exudate.

The equilibrium contact angle of L-PDMS on mosquito tarsi was calculated using the value of attractive force. The contact angle is given by.

$$\theta = \cos^{ - 1} \left( {\frac{F}{\pi D\gamma }} \right),$$

where *F* is the attractive force, *D* is the thickness of mosquito tarsus, and γ is the surface tension of the tested liquid^41^. The equilibrium contact angle of L-PDMS was obtained by using the average of the attractive force [*F* =  − 4.96 ± 0.52 (mean ± s.d., n = 8)] (Fig. 1e), and the thickness of the mosquito tarsus was calculated using images from a digital microscope (VHX-5000, Keyence Corporation) taken at a point 250 $\upmu$m from its tip [thickness, *D* = 76.1 ± 5.53 $\upmu$m (mean ± s.d., *n* = 28)].

### Dynamics of attractive capillary force on PDMS thin films using atomic force microscopy (AFM)

AFM was used to measure the attractive capillary force caused by the meniscus. A uniform PDMS film was required to accurately detect the attraction using the probe (RTESPA-150, spring constant: 6 N/m, Bruker), and PDMSs (L-PDMS and H-PDMS) were spin-coated on a silicon-wafer to form a thin film (K-359SD1 Spinner, Kyowariken Co., Ltd.) (Supplementary Table S4). To render the film thickness thinner than the size of the probe, the thickness was set to 200 nm; a 120-nm-thick film was also prepared to investigate the thickness dependence on H-PDMS. Because the PDMSs used in the mosquito contact-time test alone cannot form these thin films by spin-coating, volatile PDMS (KF96L-1cs, Shin-Etsu Chemical Co., Ltd.) was added as a solvent to reduce the viscosities. This solvent volatilised during spin coating. The thickness of the film was measured using an ellipsometer (M-2000D, JA Woollam Co.).

The probe and film were set on the AFM (MFP3D, Asylum Research) (Fig. 3b). Next, the stage where the film was placed was raised to approach the probe at 1 $\upmu$m/s, and the stage was fixed immediately after the probe connected with the liquid at the jump-in process. The state was held for 60 s, and changes in attractive force were recorded in 2-ms steps. To avoid film thickness changes owing to flowing liquid, the attractive force was measured immediately after preparing the film. The measurement was also performed using another probe for each PDMS. The deflection sensitivity of each probe was determined by bringing it into contact with a solid surface (silicone-wafer substrate), and the accurate spring constant was measured by fitting of the power spectrum to frequency using thermal motion of the lever^42^.

### Mosquito landing test on forearms

All the landing tests using humans were approved by the Ethics Committee of Kao Corporation. All the subjects provided their informed consent to participate in the experiments. Experiments were performed using human volunteers (five males aged 25–41). The test subjects wore long rubber gloves that covered their forearms from the fingers to the elbows (Singer Latex Long Glove M, Utsunomiya Seisaku Co., Ltd.) after washing their forearms with body soap (Bioré, Kao Corporation). We prepared an exposure area with dimensions of 4 cm × 5 cm on their forearms by cutting a square hole in part of the rubber glove and reinforcing the cut edges with drafting tape (Scotch, Drafting tape dimensions 18 mm × 30 m, 3 M Company). The mosquitoes could not bite the humans through the rubber surface of the glove. PDMSs (L- and H-PDMS), squalane, and glycerol were applied to the exposed area of the forearms (application ratio: 0.25 mg/cm^2^).

The forearms of each subject were placed in an insect cage (BugDorm-1, MegaView Science Co., Ltd.) and exposed to approximately 50 female mosquitoes that had been fed only water for over 12 h to ensure that they were starving. Because the liquids gradually moved around the forearm during trials, we began the experiments within 1 min after applying the liquid coating to the forearm. We recorded the biting number of the first 15 mosquitoes that attempted to land on the exposed area of the test subject forearm. A bite was scored if the proboscis was in contact with the skin, and then the mosquitoes were removed with an aspirator. Although blood-feeding is ideally confirmed by visual assessment of blood in the abdomen, if multiple mosquitoes stayed on the exposed area, it was difficult to determine the landing of the newly contacted mosquito. Instances of mosquitoes attempting to bite using parts of the surrounding tape as a foothold were excluded from biting ratio calculations. These experiments were performed at 28 °C and 70% RH.

### Hippopotamus secretion sampling

Hippopotamus secretion sampling was approved and monitored by the Animal Care Committee of Kao Corporation. We collected secretions from a male *Hippopotamus amphibious* born in 1982 living in captivity (Adventure World, Wakayama, Japan). The hippo emerged from the water at approximately 2 p.m. and spent approximately 30 min in the sunshine, during which time the secretion was produced. The weather was clear, and the temperature was approximately 29.4 °C on the day of sampling. Zoo employees wiped the hippo’s face with paper towels (Itoman Towel, L200, Itoman Co., Ltd.), and then we extracted the reddish secretion by squeezing the immersed sheet on a polystyrene dish (Iwaki, Microplate with Lid, 3,810–006, Asahi Glass Co., Ltd.). The secretion was collected using a micropipette (Eppendorf Reference 2) and was subsequently stored in micro tubes (Eppendorf Safe-Lock Tubes, 1.5 mL, Biopur). These samples were cooled at approximately 2 °C in a thermostat box (CoolBox XT/2XT, BCS-576, Brooks Life Science). After 4 h of transportation, we used the exudate (which had changed from red to brown owing to exposure to ultraviolet irradiation) in our experiments. The water content of the hippo secretion was determined using a Karl Fischer titrator (AQV-2200A, Hiranuma Sangyo Co., Ltd.). The water content was 97.247 ± 0.855% (mean ± s.e.m., *n* = 3).

### Surface tension and viscosity measurement of hippopotamus secretion

Since we could only collect samples of secretion in volumes of ~ 5 mL by wiping the face of the hippopotamus face during sampling, the exudate volume was not sufficient to evaluate the surface tension or viscosity of the secretion using the methods described previously. Thus, surface tension was evaluated by measuring its capillary action. A glass tube (DG-1, Surfgauge Instrument) was prepared, and the tip of the tube was placed on the surface of a droplet of the secretion. The liquid rose into the vertically fixed tube and the surface tension was calculated using the distance the secretion climbed up the tube and the liquid’s density. The secretion density (0.99 g/cm^3^ at 23 °C) was roughly estimated by measuring the masses of 1 mL volumes of secretion. Viscosity was measured using a rheometer (MCR302, Anton Paar). CP50 parallel plates were used for the measurements ($\phi$ = 49.969 mm, angle: 0.994°). This measurement showed the viscosity as a function of shear rate (flow curve). Flow curves resulting from three separate measurements were highly similar.

### Statistical analysis

All statistical analyses were performed using EZR^43^. The data of contact time of mosquitoes (Figs. 2c, 3a, 4e), contact angle of tested liquids (Fig. 1b), and mosquito bites (Fig. 4b) were analysed using one-way analysis of variance (ANOVA) with the Tukey post hoc test. Details of the statistical analysis are given in the figure legends. The number of times each experiment was repeated (*n*) is also indicated in the figure legends.

### Ethics statement

All mosquito landing tests using human forearms were reviewed and approved by the Ethics Committee of Kao Corporation. All subjects provided their informed consent to participate in the experiments. Hippopotamus secretion sampling was approved and monitored by the Animal Care Committee of Kao Corporation. All experiments were performed in accordance with the relevant guidelines and regulations.

## Supplementary information

Supplementary file1

Supplementary file2

Supplementary file3

Supplementary file4

Supplementary file5

Supplementary file6

## Data Availability

All data supporting the findings of this study are available in the main text or the supplementary materials. Correspondence and requests for materials should be addressed to the corresponding author (iikura.hiroaki@kao.com).
