# Supplementary material for: Mosquito repellence induced by tarsal contact with hydrophobic liquids
Source: Sci Rep. 2020 Sep 2;10:14480. doi: 10.1038/s41598-020-71406-y (PMC7468126; doi:10.1038/s41598-020-71406-y)
Supplement: Supplementary file 1 — Supplementary file1 [file 41598_2020_71406_MOESM1_ESM.pdf]

Supplementary Information

Mosquito repellence induced by tarsal contact with hydrophobic liquids

Hiroaki Iikura[1, 2]*, Hiroyuki Takizawa[3], Satoshi Ozawa[2], Takao Nakagawa[3], Yoshiaki Matsui[1, 2], Hiromi Nambu[1, 2]

[1]Material Science Research, Kao Corporation, 2-1-3 Bunka, Sumida, Tokyo 131-8501, Japan
[2]Material Science Research, Kao Corporation, 1334 Minato, Wakayama, Wakayama 640-8580, Japan
[3]Personal Health Care Products Research, Kao Corporation, 2-1-3 Bunka, Sumida, Tokyo 131-8501, Japan

*Correspondence and requests for materials should be addressed to H.I. (iikura.hiroaki@kao.com)

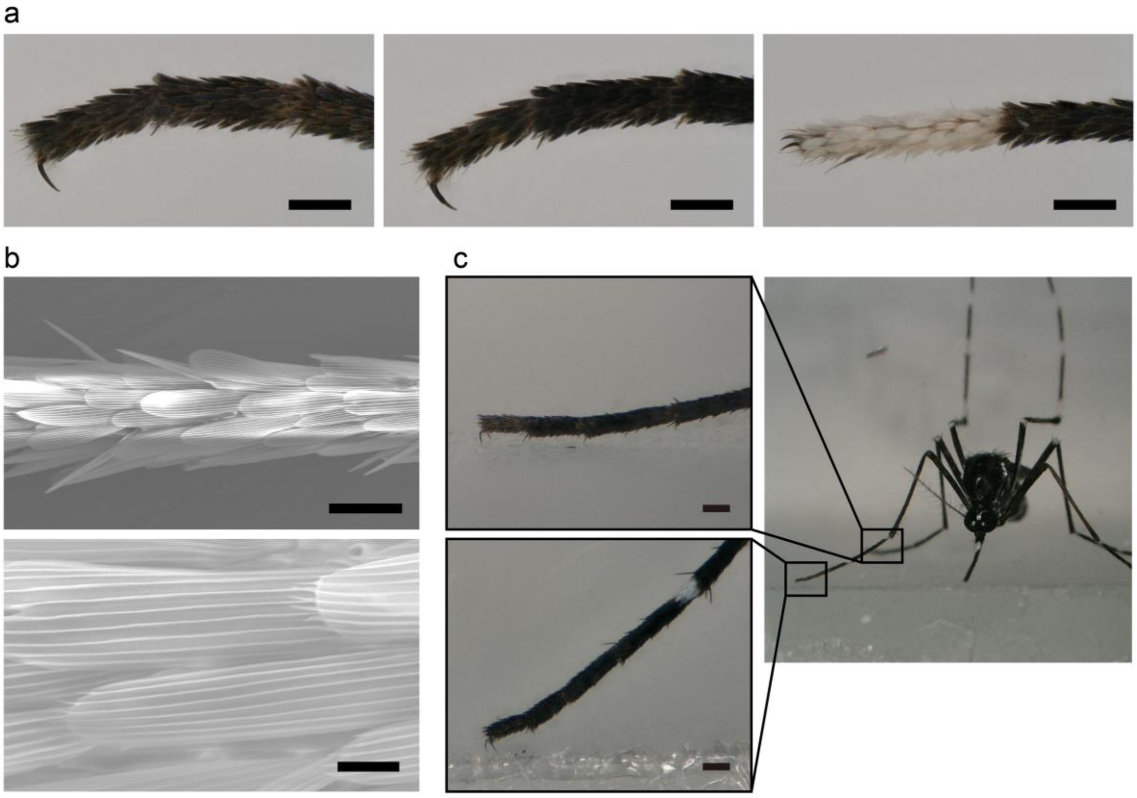

Supplementary Fig. S1 Microscopic images of mosquito legs a. Mosquito foreleg (left), middle leg (centre), and hind leg (right). Scale bars: 100 μm. **b.** Scanning electron microscopy images of scales on a mosquito foreleg. Scale bars: 50 μm (top), 10 μm (bottom). **c**. Settling-down mosquito on an uncoated ground-glass substrate. Foreleg (bottom left) and middle leg (top left). Scale bars: 100 μm.

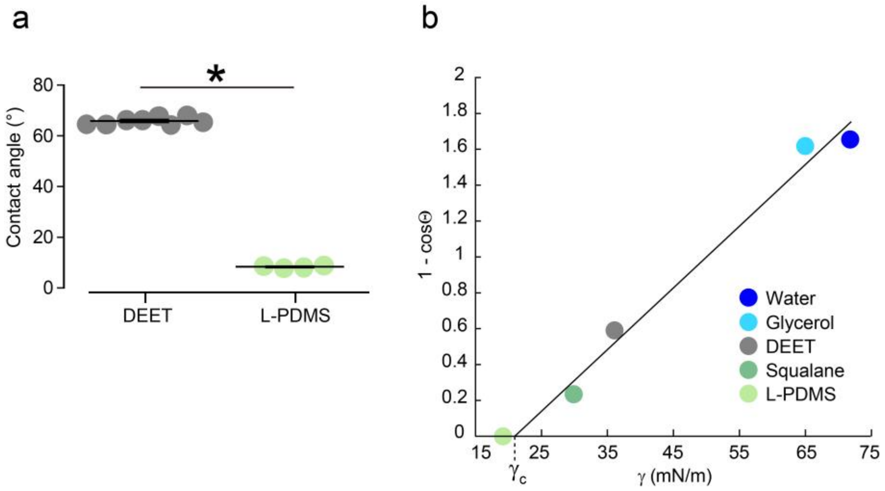

Supplementary Fig. S2 Contact angle of droplets on the scale carpet a. Contact angle of DEET and L-PDMS on the mosquito-scale carpet 1 s after liquid deposition (n = 4, 8). For DEET, the angle showed an equilibrium state, whereas the PDMS continuously spread on the surface after 1 s. The contact angle of DEET averaged 65.7° (γ = 36.1 mN/m), suggesting relatively low wettability. We anticipated that large application ratios would be required to shorten the mosquito contact-time via wetting-based repellence, implying that receptor-based mechanism likely contributes to contact-based repellence of DEET [S1]. The horizontal line represents the mean ± s.e.m. *Contact angle varied significantly between liquids (Student's t-test, $P = 10^{-15}$). **b.** Determination of the critical surface tension (γ_c) of mosquito tarsi. Contact angles, except for L-PDMS, were obtained with droplet deposition of each liquid. For the measurement with L-PDMS, the wetting did not reach equilibrium within the measurement time; thus, its contact angle was calculated using the attractive force measured with a force tensiometer (Fig. 1e). Fitting line (solid black line) was obtained with the least-square method using four plots, except for L-PDMS, because the PDMS spreads completely owing to its low surface tension (θ = 0°). Critical surface tension γ_c was 20.9 mN/m; when $\gamma < \gamma_c$, liquids spread completely on mosquito tarsi (total wetting: θ = 0°).

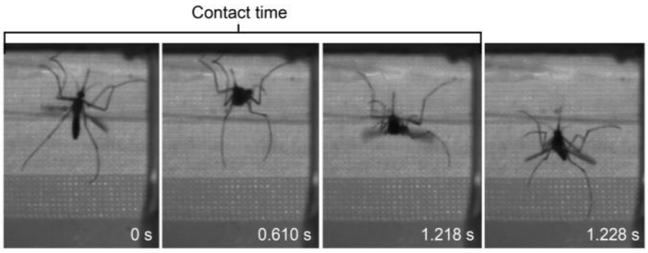

Supplementary Fig. S3 Frame-by-frame photos of mosquito on substrate coated with squalane oil. Application ratio: 0.25 mg/cm^2. Contact time was defined as the elapsed time between the recorded time when at least one tarsi or proboscis contacted the substrate and the time when all mosquito tarsi were removed.

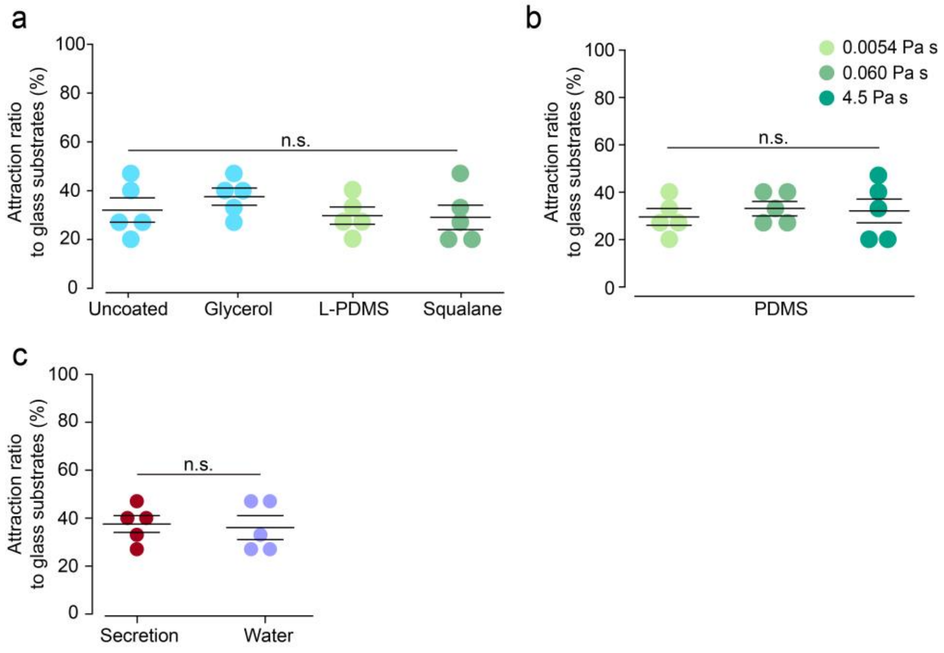

Supplementary Fig. S4 Attraction ratio to liquid-coated ground-glass substrates. **a., b.** Attraction ratio to liquid-coated glass substrate, as shown in Fig. 2c and 3a. Application ratio: 0.25 mg/cm^2. The horizontal line corresponds to the mean ± s.e.m. (n = 5). One-way ANOVA using Tukey post hoc test, (**a**): P = 0.478, (**b**): P = 0.779; n.s., not significant. **c.** Attraction ratio to ground-glass substrates coated with hippopotamus secretion and water. Application ratio: 2.0 mg/cm^2. Student's t-test, P = 0.82; n.s., not significant.

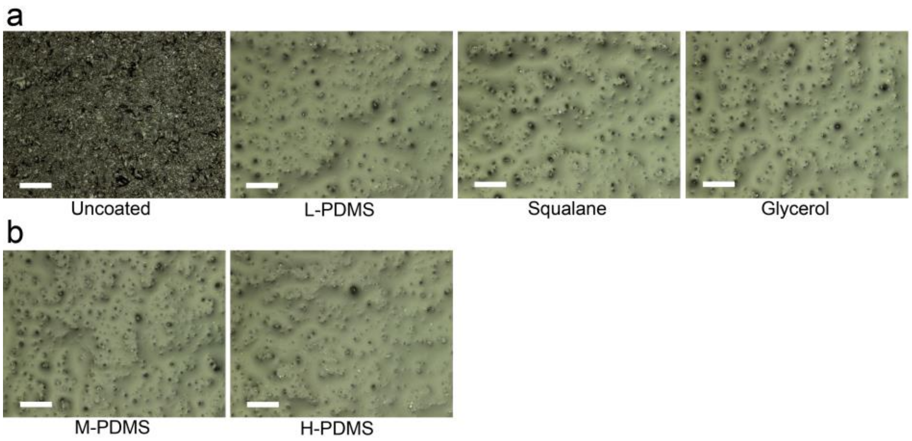

c

	Uncoated	Glycerol	L-PDMS		M-PDMS	H-PDMS		Squalane
Application ratio (mg/cm^2)		0.25	0.25	2.0	0.25	0.25	2.0	0.25
Arithmetical mean height (μm)	3.52	1.32	1.29	0.30	1.50	1.44	0.57	1.35
s.e.m	0.047	0.065	0.045	0.058	0.094	0.12	0.063	0.13
Groups	A	B	B	C	B	B	C	B

Supplementary Fig. S5 Ground-glass substrates used in mosquito contact-time test. a., b. Image of surface of uncoated glass substrate and same substrate after application of liquids specified in Fig. 2c and 3a. Application rate: 0.25 mg/cm^2. Scale bars: 100 μm. **c.** Mean height of uncoated substrate and liquid-coated substrates (n = 3). The letters (A, B, or C) indicate the significant variance among the different liquids (one-way ANOVA with the Tukey post hoc test, $P = 10^{-13}$).

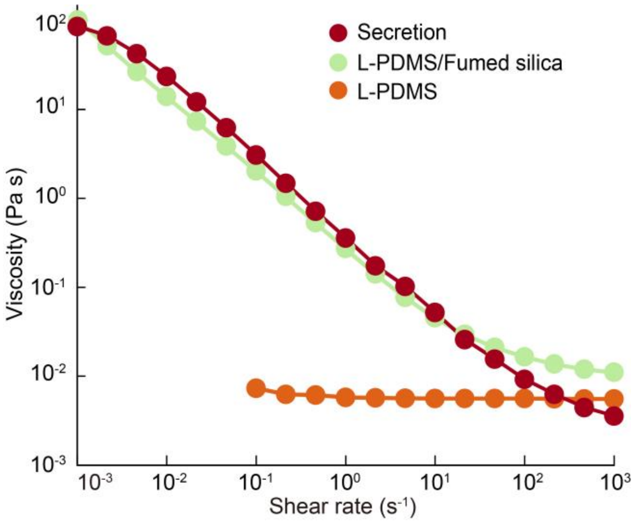

Supplementary Fig. S6 Flow curve of shear-thinning fluids.
Flow curve (viscosity as a function of shear rate) of hippopotamus secretion and fumed silica suspension in L-PDMS exhibited shear-thinning behaviour. These two curves were comparable. L-PDMS is a Newtonian fluid in which the viscosity did not vary by shear rate.

Supplementary Table S1 Surface tension (γ) and viscosity (η) of liquid samples used in this research. KF96A-6cs, KF96-50cs, and KF96-5000cs are denoted in the paper as L-PDMS, M-PDMS, and H-PDMS, respectively.

	Glycerol	Polydimethylsiloxane (PDMS)			Squalane	DEET
		KF96A-6cs	KF96-50cs	KF96-5000cs		
γ (mN/m)	65.0	19.2	20.9	21.4	29.9	36.1
η (Pa s)	0.95	0.0054	0.060	4.5	0.042	0.016

Supplementary Table S2 Contact angle of droplets on the scale carpet. The values were measured 1 s after liquid deposition.

	Water	Glycerol	L-PDMS	Squalane	DEET
Contact angle (°)	129.1	128.4	8.5	40.8	64.4
	132.6	124.8	7.7	39.5	64.3
	128.2	129.1	8.0	40.3	66.1
	139.1	133.3	8.7	39.6	66.1
	131.8	125.9		40.1	67.6
	127.7	130.1		39.6	64.2
	134.1	128.7			67.9
	124.1	124.2			65.3

Supplementary Table S3 Contact-time of mosquitoes on ground-glass substrates coated with the liquids addressed in this study. The measurement results of contact-time of each trial are displayed. The ratio (C/C ratio) of mosquitoes that made ceasing wing motions after making contact with the substrate was calculated.

	Uncoated	Glycerol	PDMS					Squalane	Secretion	Water	Suspension
γ (mN/m)		65.0	19.2	19.2	20.9	21.4	21.4	29.9	25.1	71.8	18.9
η (Pa s)		0.95	0.0054	0.0054	0.060	4.5	4.5	0.042			
Application ratio (mg/cm^2)		0.25	0.25	2.0	0.25	0.25	2.0	0.25	2.0	2.0	0.25
C/C Ratio (%)	76.9	83.3	33.3	0	66.7	69.2	58.3	66.7	21.4	90.0	41.7
Contact time (s)	0.018	0.046	0.006	0.028	0.020	0.020	0.056	0.022	0.018	0.032	0.014
	0.024	0.048	0.016	0.040	0.028	0.046	0.10	0.030	0.024	2.9	0.028
	0.032	10	0.018	0.048	0.028	0.048	0.11	0.048	0.028	5.1	0.054
	14	14	0.038	0.056	0.036	0.056	0.16	0.048	0.038	6.1	0.056
	19	22	0.040	0.058	1.8	2.5	0.54	1.2	0.038	17	0.058
	35	37	0.066	0.060	5.7	16	0.66	1.6	0.040	20	0.12
	38	47	0.094	0.084	7.0	27	1.9	3.9	0.042	23	0.38
	50	64	0.14	0.18	7.2	29	2.0	8.9	0.042	27	1.8
	77	69	1.3		7.5	31	4.0	19	0.064	52	1.8
	86	96	1.9		16	40	4.6	26	0.092	77	2.0
	105	133	2.5		25	55	5.5	39	0.19		2.2
	130	151	3.0		25	60	10	44	0.23		3.4
	145					61			7.8		
									16		

Supplementary Table S4 Preparation conditions and film thickness of spin-coated PDMSs on a silicon-wafer used in AFM measurement.

	L-PDMS	H-PDMS	
Concentration (wt%)	8.0	5.0	5.0
Spin speed (rpm)	2000	2000	6000
Film thickness (nm)	201	202	122

Supplementary Video Captions

Supplementary Video S1 Female mosquito emergence. This video is played at 8 times actual speed.

Supplementary Video S2 Mosquito landing on a skin replica substrate. This video is played at 0.03 times actual speed.

Supplementary Video S3 Mosquito contacting on a ground-glass substrate coated in squalane. Application ratio: 0.25 mg/cm^2. This video is played at 0.06 times actual speed.

Supplementary Video S4 Meniscus formation on a mosquito tarsus. Application ratio of M-PDMS: 2 mg/cm^2. This video is played at 0.03 times actual speed.

Supplementary Video S5 Mosquito contacting on a ground-glass substrate coated with L-PDMS. Application ratio: 0.25 mg/cm^2. This video is played at 0.06 times actual speed. The mosquito escaped from the substrate without ceasing wing motion.

Reference

S1. Dennis, E. J., Goldman, O. V. & Vosshall, L. B. *Aedes aegypti* mosquitoes use their legs to sense DEET on contact. *Curr. Biol.* **29,** 1551-1556 (2019).
